# Endothelin-1 receptor blockade impairs invasion patterns in engineered 3D high-grade serous ovarian cancer tumouroids

**DOI:** 10.1042/CS20240371

**Published:** 2024-11-15

**Authors:** Judith Pape, Umber Cheema, Piera Tocci, Rosanna Sestito, Ilenia Masi, Marilena Loizidou, Anna Bagnato, Laura Rosanò

**Affiliations:** 1 UCL Division of Surgery and Interventional Science, London, U.K.; 2 Unit of Preclinical Models and New Therapeutic Agents, IRCCS, Regina Elena National Cancer Institute, Rome, Italy; 3 Institute of Molecular Biology and Pathology (IBPM), National Research Council (CNR), Rome, 00185, Italy

**Keywords:** endothelins, ovarian cancer, stroma, tumoroid

## Abstract

High-grade serous ovarian cancer (HG-SOC), accounting for 70–80% of ovarian cancer deaths, is characterized by a widespread and rapid metastatic nature, influenced by diverse cell types, cell–cell interactions, and acellular components of the tumour microenvironment (TME). Within this tumour type, autocrine and paracrine activation of the endothelin-1 receptors (ET-1R), expressed in tumour cells and stromal elements, drives metastatic progression. The lack of three-dimensional models that faithfully recapitulate the unique HG-SOC TME has been the bottleneck in performing drug screening for personalized medicine. Herein, we developed HG-SOC tumouroids by engineering a dense central artificial cancer mass (ACM) containing HG-SOC cells, nested within a compressed hydrogel recapitulating the stromal compartment comprising type I collagen, laminin, fibronectin, and stromal cells (fibroblasts and endothelial cells). ET-1-stimulated HG-SOC cells in the tumouroids showed an altered migration pattern and formed cellular aggregates, mimicking micrometastases that invaded the stroma. Compared with control cells, ET-1-stimulated tumouroids showed a higher number of invasive bodies, which were reduced by treatment with the dual ET-1 receptor (ET-1R) antagonist macitentan. In addition, ET-1 increased the size of the invading aggregates compared with control cells. This study establishes an experimental 3D multicellular model eligible for mechanical research, investigating the impact of matrix stiffness and TME interactions, which will aid drug screening to guide therapeutic decisions in HG-SOC patients.

## Introduction

Ovarian cancer is the most aggressive gynecological malignity and the fifth most common cause of cancer deaths worldwide [1], with high-grade serous ovarian cancer (HG-SOC) being responsible for more than 85% of OC. Because of the lack of an effective way of screening and the presence of uncertain symptoms, patients are usually diagnosed at an advanced stage after metastasis occurs, mainly via the transcoelomic route [1,2].

The metastatic process is heavily influenced by the extracellular matrix (ECM) composition, mechanical cues, and the interactions of cancer cells with the cellular components of the surrounding tumour microenvironment (TME), responding to signals provided by the TME, such as growth factors, to facilitate the metastatic spread [3]. In this context, within the G-protein coupled receptor (GPCR) family, the endothelin-1 has a critical role in HG-SOC progression, by controlling different tumour-promoting effects via the receptors ET_A_ (ET_A_R), and ET_B_ (ET_B_R) [4,5]. Specifically, the majority of tumour-promoting events are regulated via ET_A_R, while ET_B_R appears to mainly be involved in TME-associated functions [5]. According to these findings, the emerging approach for HG-SOC treatment is to block tumour/stroma communication with dual ET-1 receptor (ET-1R) antagonists, such as macitentan [6–9].

Several *in vitro* and *in vivo* studies provide evidence that the activation of the ET-1R axis has been implicated in conferring features associated with the acquisition of aggressive traits in HG-SOC [10]. In this context, by integrating proteolytic and adhesion signaling, the ET-1 axis fine-tunes HG-SOC cell invasion and metastasis by cytoskeleton remodelling and formation/activation of proteolytic structures, invadopodia, modifying the stroma to a permissive state to invasion and participating in the establishing of the pre-metastatic niche [8,9,11,12]. ET-1 rewires the behaviour of stromal elements, such as fibroblasts, mesothelial, and endothelial cells, to generate a corrupted TME [6,13,14]. These interactions allow cancer cells to survive and escape from therapeutic drugs.

Recent developed 3D HG-SOC models maintain features of tumour tissue architecture observed *in vivo* and can predict response to radiation and chemotherapy treatments [15]. However, the lack of TME components precludes the development or evaluation of emerging tumour/TME-targeted therapies. Thus, the use of a biomimetic tumouroid model which consists of a relatively hypoxic central cancer mass surrounded by its microenvironment (stroma) has not yet been explored to understand the HG-SOC-TME crosstalk and for the evaluation of ET-1R blockade in tumour growth and invasive process.

Complex tumouroids can mimic the heterogeneity of solid cancer tissues, by including both cancer masses and the surrounding stroma and incorporating stromal cells, such as fibroblasts and endothelial cells, in a stiffness-modulable matrix scaffold. Several studies demonstrated that tumouroids represent a very useful *in vitro* platform to evaluate cancer progression and metastasis and the impact of the TME on drug effectiveness [16–19]. In the present study, we aimed to use this model to investigate the impact of ET-1 axis in HG-SOC tumouroids and to test the feasibility of ET_A/B_R blockade with macitentan in the invasion pattern.

## Materials and methods

### Cell cultures

Ascites patient-derived HG-SOC primary cells (PMOV10), carrying a TP53 germline missense mutation variant (R337T) on exon 9, which closely recapitulate the genomic traits, the histopathology and the molecular features of the HG-SOC patient (stage III, age 69), were established, characterized, and maintained as previously described [7]. The protocols for ascitic sample collection along with clinical information were approved by the Regina Elena institutional review board (IRB) and HG-SOC patients provided written informed consent. The purity of PMOV10 primary cells was assessed by immunophenotyping with a panel of moAbs (including OCT-125, WT1, calretinin and keratin 7) recognizing ovarian cancer-associated antigens. Established human ovarian serous adenocarcinoma cell line, HEY (American Type Culture Collection, LGC Standards, Teddington, UK), Human dermal fibroblasts (HDFs) and Human umbilical vein endothelial cells (HUVECs) (provided by the UCL Division of Surgery and interventional Sciences) were cultured under routine conditions in Dulbecco's modified Eagle medium (DMEM/F-12, low glucose, Sigma Aldrich,St. Louis, MO, USA), High glucose DMEM (Thermo Fisher Scientific, Hemel Hempstead, UK) and Endothelial cell growth medium with supplements (PromoCell, Heidelberg, Germany), respectively. The cell culture media were supplemented with 10% heat-inactivated fetal bovine serum (FBS, Thermo Fisher Scientific) and 1% penicillin (5,000 units/mL) and streptomycin (5,000 µg/mL) (Thermo Fisher Scientific). ET-1 was purchased from Bachem (Bubendorf, Switzerland) and it was used at 100 nM and incubated with the cells for the indicated times. Macitentan, also called ACT-064992 or N-(5-[4-bromophenyl]-6-pyrimidine-4-yl)-N'-propylsulfamide, was kindly provided by Actelion Pharmaceuticals Ltd (Allschwil, Switzerland), and was used at a concentration of 1 µM for 30 min before the addition of ET-1.

### Metabolic assays

Lactate dehydrogenase (LDH) activity was measured in PMOV10 cells by using an LDH microplate assay kit (Cohesion Biosciences, London, UK), according to the manufacturer's instruction. Briefly, 3 × 10^5^ cells were seeded in 60-mm culture dishes and, after 24 h in serum-free medium, were stimulated with ET-1 (100 nM) alone or in combination with macitentan (1µM) for 48 h. Cells were then lysed and transferred in 96-well plates in triplicate together with the substrates provided by the kit. LDH activity was measured at 450 nm with the Multiskan FC instrument (Thermo Fisher Scientific) and was expressed as unit of enzymatic activity normalized to the number of cells. In parallel, conditioned media (CM) were collected and 50 µl of each sample were transferred in 96-well plates in triplicate. The lactate content was measured at 570 nm with the Multiskan FC instrument by using a lactate assay kit (Sigma-Aldrich), as suggested by the manufacturer.

### Tumouroid manufacture

To create tumouroids as previously described [18,19], patient-derived HG-SOC cells and HEY cells were firstly incorporated into an artificial cancer mass (ACM). Briefly, monomeric collagen type I of rat tail origin at 2 mg/mL (First Link, Birmingham, UK) was mixed with 10× MEM (Thermo Fisher Scientific) and neutralizing agent (N.A.), according to the RAFT™ protocol. The desired cellular concentration, in this case, 5 × 10^4^/ACM, was added into a set volume of DMEM to the collagen/10× MEM/N.A. mix. To cross-link the collagen, 240 µL of the cellular collagen mix was suspended into a 96-well plate and incubated at 37°C for 15 min. Plastic compression was then utilized to withdraw the water content from these hydrogels using the 96-well size RAFT™ absorbers (Lonza, Slough, UK) for 15 min. This increased the collagen density to 10%. For the stromal compartment, the ACMs were placed into a 24-well plate containing 1.3 mL of the non-cross-linked collagen mix. Laminin at 50 µg/mL, fibronectin at 25 µg/mL, as well as 25,000 HDFs/sample and 100,000 HUVECs/sample were added. This whole tumouroid was then again be left to cross-link at 37°C for 15 min and subsequently plastic-compressed using the 24-well RAFT™ absorbers for 15 min at room temperature. These full tumouroids were then be cultured for up to 21 days in 1.0 mL of cell-appropriate media (mix 1:1:1 if necessary) at 5% carbon CO_2_ atmospheric pressure and 37°C with a 50% media change every 48 h.

### Cell viability assay

The viability of ACMs and tumouroids was determined using the alamarBlue™ assay (Invitrogen, Thermo Fisher Scientific). Briefly, 48 h after light illumination, alamarBlue™ dye solution (10% of the total volume in culture medium) was added to the wells and incubated at 37°C for 4 h. Afterwards, the supernatant from each well was transferred into black 96-well well plates in triplicates. Fluorescence was measured following excitation at 530 nm and emission was detected at 620 nm using a fluorescence plate reader (Fluoroskan™ Ascent, Thermo Scientific). The alamar blue assay fluorescence readings would have been normalized to a media control.

### Immunostaining

At the end of the experiments, tumouroids were formalin-fixed for 1 h with 10% neutrally buffered formalin. The samples were then washed twice with PBS. The constructs were permeabilized using PBS containing 1% bovine serum albumin (BSA) and 0.2% Triton X-100 for one hour at room temperature (both Sigma-Aldrich). The tumouroids were stained using Anti-Cytokeratin 7 (CK7) antibody (Abcam, Cambridge, UK) (for HEY cells), Anti-Vimentin antibody (Santa Cruz, Dallas, USA) (for HDFs), Anti-CD31 antibody (Abcam, Cambridge, UK) (for HUVECs) and Anti-MMP14 (Abcam, Cambridge, UK) (for invasion and matrix degradation). All primary antibodies were mixed in the 1% BSA and 0.2% Triton X solution at a 1/200 ratio and incubated at 4°C overnight. The antibody solution was removed, and tumouroids were washed three times with PBS each time incubating at room temperature for 5 min. Secondary antibodies (Alex Fluor 488 or Alex Fluor 594, both Abcam) were added at a 1/1000 ratio within the same 1% BSA and 0.2% Triton X solution and incubated for 2.5 h at room temperature. The antibody solution was then again removed and the tumouroids were washed three times with PBS for 15 min. Finally, nuclei were stained by adding NucBlue™ (Invitrogen, Thermo Fisher Scientific) straight to the well, and constructs were imaged using the Zeiss AxioObserver with ApoTome.2 and Zeiss ZEN software (Zeiss, Oberkochen, Germany).

### Image analysis for invading bodies

The images were analysed using open-source Image J software to measure the distance within the stroma that was invaded by cancer cells in simple tumouroids following ET-1 stimulation and/or Macitentan treatment. For each tumouroid, four measurements were taken from the edge of the original cancer mass to the farthest cancer cell cluster within the stroma in a clockwise manner (12, 3, 6, and 9 o'clock). Each condition had three tumouroid repeats, generating 12 measurements per condition from which averages were taken.

### Statistical analysis

All analysis and statistics were conducted in GraphPad Prism 8 software. Results were shown as means ± standard deviation (SD) of experiments performed in triplicates for all described conditions. Values of *P*<0.05 were considered statistically significant.

## Results

### Metabolic activity in HG-SOC tumouroids

**Figure 1:**
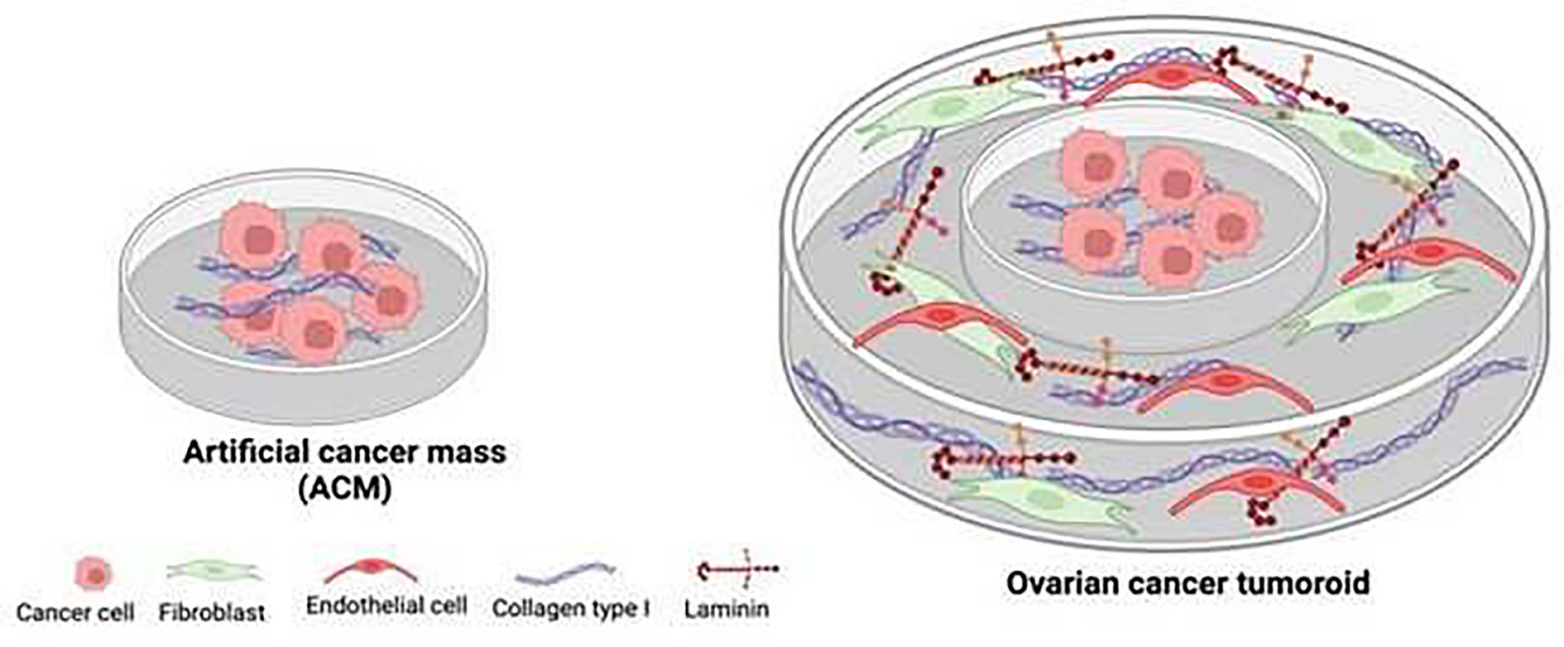
Schematic representation of artificial cancer mass (ACM) and tumouroid model with a complex stroma Each ACM, in which ovarian cancer cells were inserted into a cellular added to collagen type I, was incorporated in a complex stroma, Insertion of the ACM into a cellular collagen type I/ laminin hydrogel populated with fibroblasts and endothelial cells. Created with BioRender.com (Agreement number CJ26WV7Q9R).

To address the aim of studying the ET-1 axis in ovarian tumouroids, we started using an ACM model containing HEY cells and in parallel a model containing patient-derived HG-SOC cells, both cultured in a 3D matrix composed of collagen type I and a patented and semi-automated process, based on previously reported UCL patent to compress the cell populated hydrogel [20] (Figure 1).

**Figure 2:**
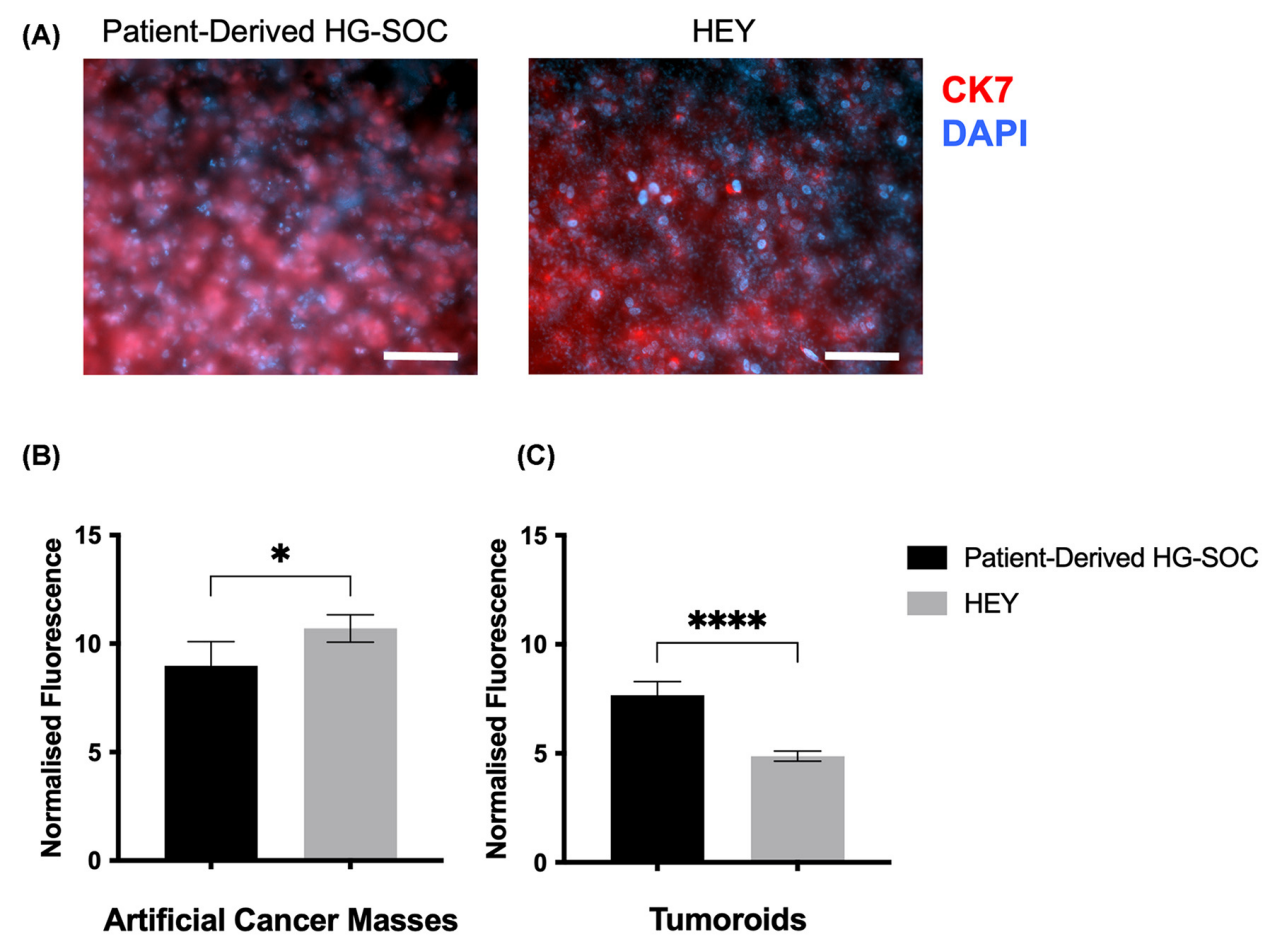
Metabolic activity in HG-SOC tumouroids (**A**) Representative ACM containing ovarian cancer cells stained for CK7 (red) and DAPI (blue). Scale bar = 50 µm. Metabolic activity of the cells within (**B**) ACMs or (**C**) tumouroids by day 14 evaluated via alamarBlue™ assay. Significance signifies unpaired t test P value.

**Figure 3:**
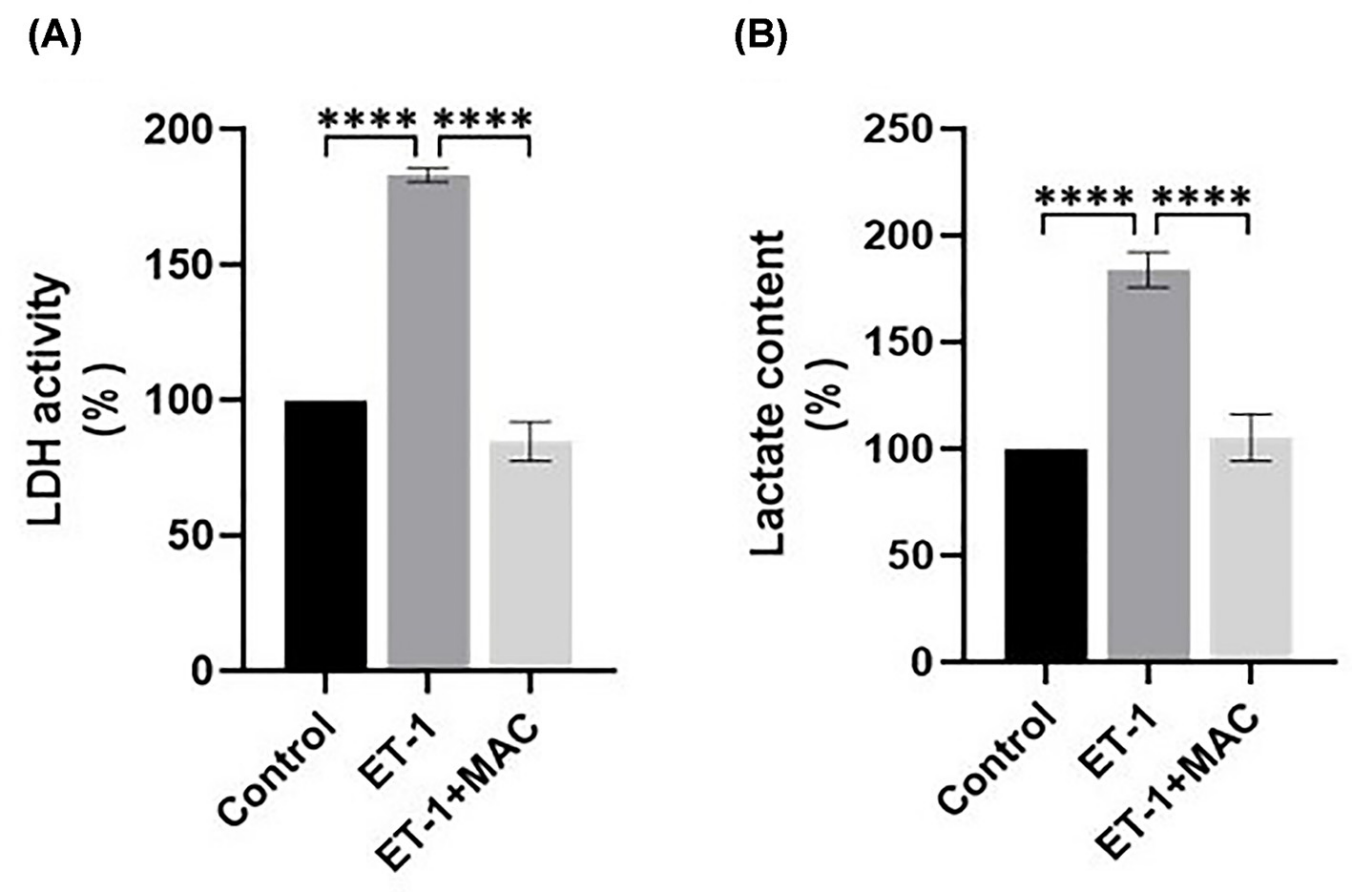
ET-1 receptor blockade impairs metabolic activity in HG-SOC cells (**A**) Lactate dehydrogenase (LDH) activity was measured in patient-derived HG-SOC cells stimulated for 48 h with ET-1 (100 nM) alone or with macitentan (1µM, MAC). Values are the mean ± SD. (**B**) The lactate content was measured in the secretome of HG-SOC cells stimulated as in A. Significance signifies ordinary one-way ANOVA P value with Tukey's multiple comparison test.

As preclinical relevant models we used ascites patient-derived, early passage primary cultures of HG-SOC cells, which recapitulate the HG-SOC features [7]. IF analysis using CK7 which is specifically expressed in epithelial cells showed the cell morphology in both ACMs (Figure 2A). Cell survival is crucial for future drug assays within the tumouroids. To establish HG-SOC cell survival and measure proliferation within the simple compartmentalized tumouroids, a metabolic assay was performed by using the alamarBlue™ cell viability assay and quantitative measurements. Initially, this assay was performed on ACMs showing significantly higher viability in the HEY cells (*P* = 0.0356) (Figure 2B). However, when in the presence of a cellular stroma, which incorporates human dermal fibroblasts and endothelial cells, patient-derived HG-SOC tumouroids had significantly higher viability (*P*<0.0001) than those with HEY cells (Figure 2C), indicating the feasibility of using patient-derived cells to develop engineered tumouroids. Indeed, by using primary HG-SOC cells, we observed that they retained high metabolic activity for 21 days when cultured with stromal cells compared with the HEY cell line. In parallel, we evaluated the metabolic activity of patient-derived HG-SOC cells treated with ET-1 regarding lactate dehydrogenase (LDH) activity and lactate production. Interestingly, while ET-1 (100 nM) significantly enhanced the LDH activity (Figure 3A, *P*<0.0001), as well as the lactate release (Figure 3B, *P*<0.0001) in the cells, this effect was strongly affected upon pharmacological ET-1R blockade with the dual ET_A_R/ET_B_R antagonist macitentan (1 µM) (Figure 3A,B, *P*<0.0001). These findings showed the efficacy of macitentan in inhibiting metabolic activity in patient-derived HG-SOC cells.

### ET-1R blockade by macitentan impairs stromal invasion in ovarian tumouroids

**Figure 4:**
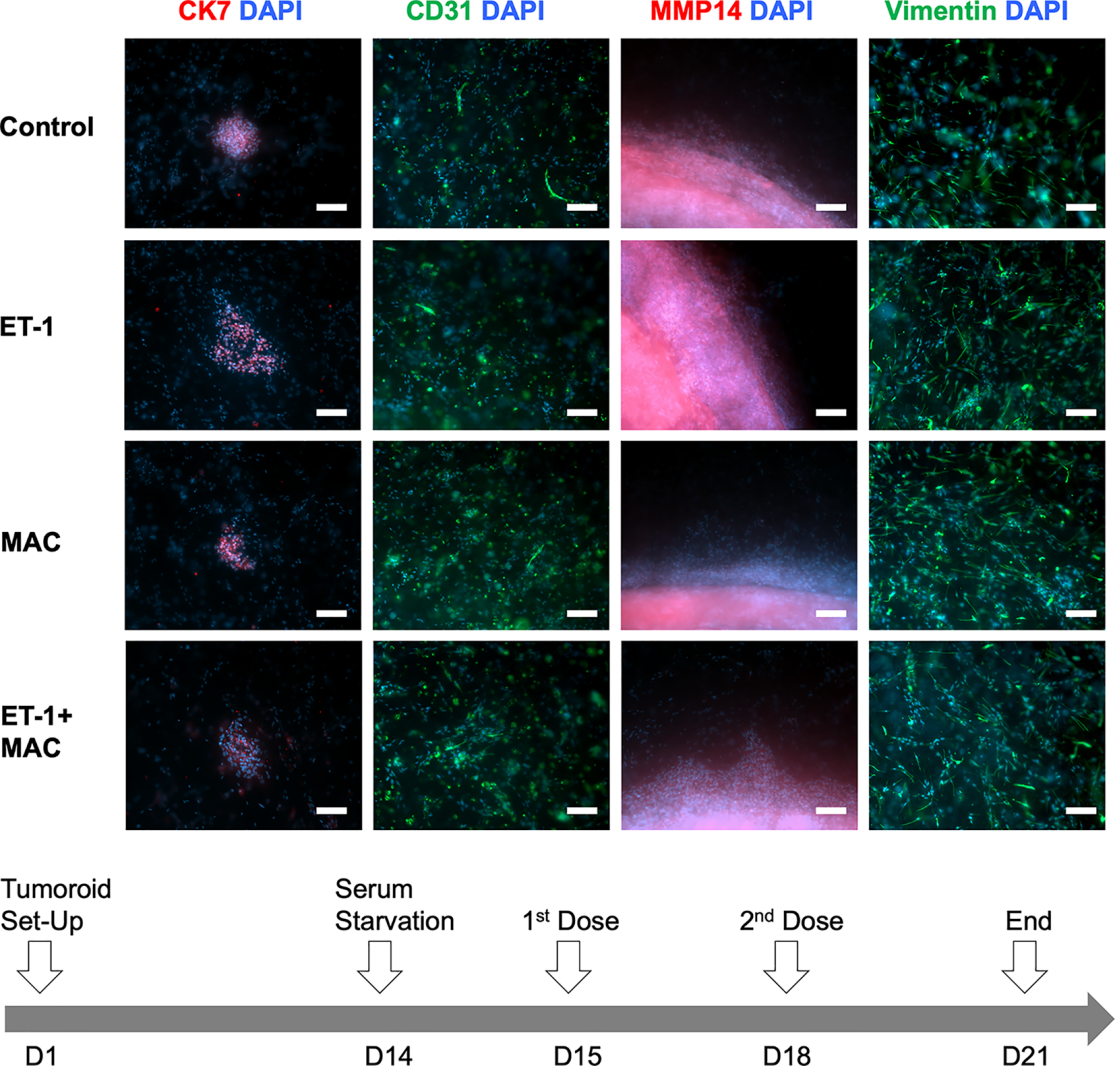
Ovarian cancer cells invade the tumouroid stroma Engineered ACMs consisting of 10% (w/v) collagen and HEY cells embedded within a stromal compartment of the same collagen density and stromal cells (HDF and HUVECs). Representative invasive bodies, fibroblasts, and endothelial structures adjacent to the invasive front of HEY biomimetic tumouroids. Drug treatment with ET-1 (100 nM) and/or macitentan (MAC) (1 µM). HEY cancer cell morphology was assessed using immunofluorescence of CK7 (red) and MMP14 (red) and DAPI (blue), HDF morphology using vimentin (green) and DAPI (blue), and HUVEC morphology using CD31 (green) and DAPI (blue). Scale bar = 100 µm.

We engineered ACMs containing HEY cells embedded in a 10% collagen matrix within stromal compartments, including dermal fibroblasts, and endothelial cells, to form tumouroids. Tumouroids were left to mature for 14 days before drug treatment. A 24 h serum starvation occurred between days 14 and 15 and then two consecutive 72 h drug treatments occurred. Drug treatments were either stand-alone ET-1 or macitentan or combination treatment. After 21 days, the samples would either be fixed for staining or analyzed for cell viability. Figure 4 shows a tumouroid consisting of all three cell types, with cancer cells stained for CK7, endothelial cells stained for CD31, and fibroblasts stained for vimentin. By day 14, the cancer cells induced by ET-1 invaded the stroma both in the area adjacent to the central cancer mass and further into the stroma. Interestingly most of the cancer cells grew mainly at the outer edges of the central cancer compartment (Figure 4). The invading cells were also characterized by the expression of MMP-14, which is considered a master matrix metalloprotease linked to the invasive behaviour of ovarian cancer cells.

**Figure 5:**
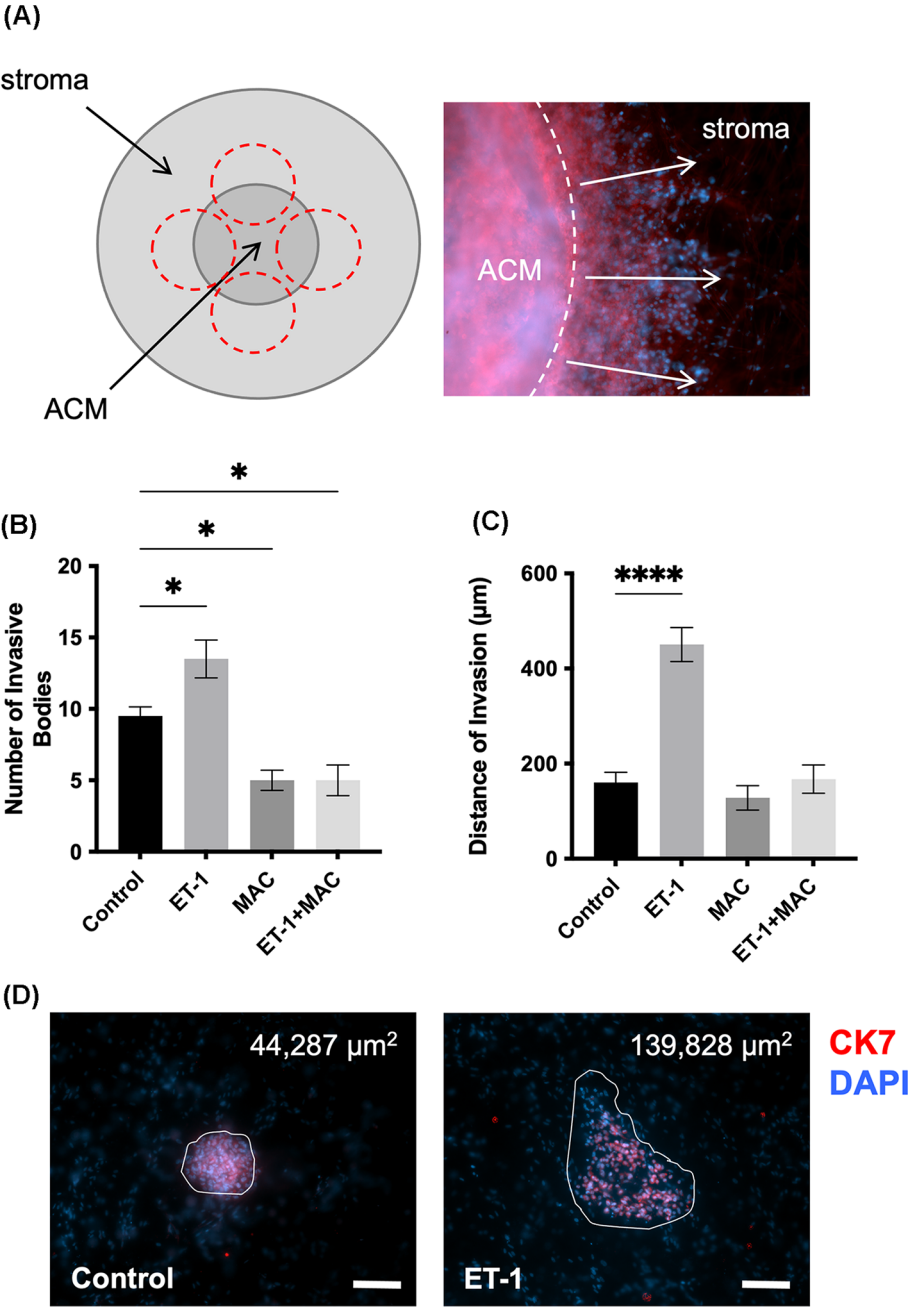
ET-1R activation enhances the number, distance and size of invasive bodies (**A**) Model of invasion patterns near the ACM. (**B**) Number of invasive bodies and (**C**) distance of invasion into stromal compartment after drug treatment with ET-1 (100 nM) and/or macitentan (MAC) (1 µM) by day 14. (**D**) Example of surface area of invasive bodies for the ET-1 treatment and control group, showing a 3-fold up-regulation after ET-1 treatment. Invasive bodies were stained for CK7 (red) and DAPI (blue). Scale bar = 100 µm. Significance signifies ordinary one-way ANOVA P value with Dunnett's multiple comparison test.

Multiple parameters were assessed to provide an in-depth understanding of invasive behaviour in 3D tumouroid models, including the count, invasive distance, and surface area of invasive bodies. The number of invasive bodies formed by HEY cells after 21 days in a 3D collagen matrix and their distance invaded the cellular stroma can be seen in Figure 5A. The number of invasive bodies was significantly up-regulated in tumouroids stimulated with ET-1 (*P*=0.0346) when compared with control conditions. At the same time, the addition of macitentan significantly inhibited this effect when alone (*P*=0.0180) or in combination with ET-1 (*P*=0.0180) (Figure 5B). This was further validated with the distance of invasion measured from the ACM (Figure 5C). As observed in Figure 5D, the size of invasive bodies under the stimulation with ET-1 was significantly increased (*P* =<0.0001), suggesting the proliferative effects of ET-1 favouring the regrowth of invasive bodies.

## Discussion

Although HG-SOC detection and advanced treatment strategies have increased in the last few decades, understanding the complex communication between cancer cells and the cells within their microenvironment still represents severe clinical challenges associated with the HG-SOC survival rate [21,22]. This highlights the need for an adequate platform to address tumour recurrence and develop new therapeutic strategies in HG-SOC treatment [23–25].

Among the complex and multifactorial determinants of the lack of major therapeutic advances in HG-SOC, the paucity of clinically relevant human models plays a critical role. One of the main causes of this failure is associated with the use of 2D models *in vitro*, which although having been established from late-stage disease, are affected by long term culture adaptation, genetic drift, and the absence of a complex *in vivo* microenvironment [24]. This can result in the loss of key cellular signalling pathways and changes in cell responses to biochemical and biomechanical stimuli, thus limiting the applicability of these models. For this reason, many efforts have been made to develop new models, such as multicellular 3D *in vitro* systems, to overcome these limitations and bridge the gap between *in vitro* studies and clinical situations. In this context, tissue engineering approaches have been used to build 3D human cancer tissues reproducing mechanical/biochemical cues that are crucial for cancer development, such as cell–cell/cell–ECM interactions and tissue stiffness, with the potential to improve our knowledge of cancer progression and enable the development of cancer treatments [23].

The tumouroid models, 3D models fabricated through plastic compression of type I collagen hydrogel embedded with cancer and stromal cells, have been revealed as very powerful engineered 3D models to recapitulate the appropriate *in vivo* TME, with the opportunity to study cancer invasion in a longitudinal manner and to investigate drug response in a physiologically relevant 3D matrix [16–18,20,26–29]. In ovarian cancer tumouroids, the efficacy of the cytotoxin Saporin was assessed using HEY ovarian cancer cells in the central cancer compartment. This study showed that ovarian tumouroids, functioning as tissue mimics, are suitable models for investigating multicellular events following a pharmacological assault [19].

In this explorative study, establishing an HG-SOC tumouroid model with a complex stromal compartment, we were able to validate the role of the ET-1 axis in the invasion and growth of this tumour. In addition, conducting macitentan drug testing validated the model as a potential candidate for future drug screening and patient-specific modelling. These ovarian tumouroids are suitable models for interrogating multicellular events following combinatorial pharmacological approaches.

Our model not only tests the ET-1 axis and HG-SOC cells within a 3D setup containing a tunable ECM as well as the tumour stroma but validates the possibility of using HG-SOC cells to study tumour/ECM and tumour/TME interactions for the advancement of patient-specific personalized medicine. In this emerging area of research, the data we present reflect the interplay between cancer cells and the stromal cells within the tumour microenvironment. Cancer cells in 2D culture have a fast-doubling time which is significantly reduced when the same cells are cultured in 3D in bio-mimetic conditions. The current study shows that where stromal cells are added to compartmentalized tumour-stroma culture, the tumouroids, there is a reduction in normalized/overall metabolism in the HEY tumouroids, and this is likely to reflect the *in vivo* scenario.

In the complex ovarian tumouroids, ET-1 treatment significantly increased the number and surface area of invasive bodies from the cancer mass into the neighbouring stroma. Moreover, this model showed how ET-1 supported an increase in the distance of budding invasive bodies from the ACM into the complex stroma. This might be due to the enhanced activity of MMPs, such as MMP14 (MT1–MMP), as evident from the localization of MMP14 at the invasive front and bodies. According to previous studies demonstrating a direct link between ET-1 and MMP14 in directing ovarian cancer cell invasion [11], it is likely that the up-regulation/activation of MMP14 driven by ET-1 is responsible for the enhanced invasion of HG-SOC cells in the tumouroids.

Considering the relevance of immune compartment in HG-SOC ecosystem, encompassing immune cells in the HG-SOC tumouroids could further advance its capacity to provide important insight that could predict clinical responses in HG-SOC patients. For future more comprehensive and dynamic drug testing studies, 3D models incorporating essential elements of the pre-metastatic niche, including the immune system, will probably lead to more tailored therapies.

From a translation point of view, results from this work indicated that in ovarian cancer tumouroids, ET-1R blockade with macitentan significantly reverted ET-1 effects, inhibiting the invasive and metastatic potential of cancer cells. These findings are consistent with our previous findings highlighting how macitentan simultaneously affects tumour and stromal compartments in HG-SOC [6], and supports the idea that ET-1R blockade could be combined with other drugs, targeting the interplay between cancer cell populations within the metastatic niche. Overall, the results of our study are promising since these ovarian tumouroids encapsulated within a stiffened 3D network matrix and co-cultured with stromal elements represent a sophisticated viable system for studying mechanosensing cancer cells interacting within their microenvironment. These findings unravel the complexities of HG-SOC ecosystem for combinatorial drug exploration in ovarian cancer research and therapeutic development.

## Clinical perspectives

- Developing 3D models that mimic the complex architecture and physiological circumstances of HG-SOC is crucial for advancing our understanding of the malignancy. The complicated interactions between different cell types and drug response can be studied by using HG-SOC tumouroids by integrating components of the tumour microenvironment, such as the extracellular matrix, endothelial cells, and fibroblasts.
- The 3D tumouroids model is a useful tool for studying the ET-1-driven tumour/TME signalling towards a pro-invasive pattern and evaluating treatment modalities by using ET-1R antagonist.
- The model system of patient-derived HG-SOC tumouroids cultured using a stiffness-modulable matrix scaffold can represent a suitable tool for the rapid development of personalized treatment strategies and accelerated drug screening.

## Data Availability

Data are available upon reasonable request to the corresponding authors.

## Abbreviations

ACM: artificial cancer mass
BSA: bovine serum albumin
CK7: cytokeratin 7
CM: conditioned media
ECM: extracellular matrix
ET-1R: endothelin-1 receptors
GPCR: G-protein coupled receptor
HG-SOC: high grade-serous ovarian cancer
LDH: lactate dehydrogenase
TME: tumour microenvironment
